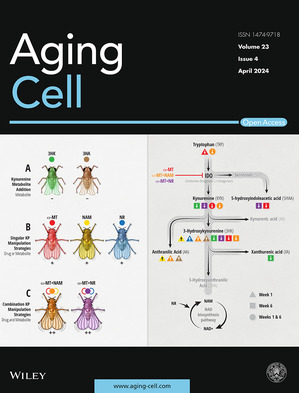# Featured Cover

**DOI:** 10.1111/acel.14179

**Published:** 2024-04-15

**Authors:** Mariann M. Gabrawy, Reyhan Westbrook, Austin King, Nick Khosravian, Neeraj Ochaney, Tagide DeCarvalho, Qinchuan Wang, Yuqiong Yu, Qiao Huang, Adam Said, Michael Abadir, Cissy Zhang, Pratik Khare, Jennifer E. Fairman, Anne Le, Ginger L. Milne, Fernando J. Vonhoff, Jeremy D. Walston, Peter M. Abadir

## Abstract

Cover legend: The cover image is based on the Research Article *Dual treatment with kynurenine pathway inhibitors and NAD+ precursors synergistically extends life span in Drosophila* by Mariann M. Gabrawy et al., https://doi.org/10.1111/acel.14102 Image Credit: Jennifer E. Fairman, CMI, FAMI, © JHUAAM.